# User testing of an adaptation of fishbone diagrams to depict results of systematic reviews

**DOI:** 10.1186/s12874-017-0452-z

**Published:** 2017-12-12

**Authors:** Gerald Gartlehner, Marie-Therese Schultes, Viktoria Titscher, Laura C. Morgan, Georgiy V. Bobashev, Peyton Williams, Suzanne L. West

**Affiliations:** 1 RTI International, 3040 East Cornwallis Rd, Research Triangle Park, Durham, NC 27709 USA; 2 Department for Evidence-based Medicine and Clinical Epidemiology, 3500 Krems, Austria; 3 Department of Maternal and Child Health, Gillings School of Global Public Health, University of North Carolina at Chapel Hill, Chapel Hill, NC 27599 USA

**Keywords:** Evidence summary, Fishbone diagram, Systematic review, Visualization, Summary of findings, User testing

## Abstract

**Background:**

Summary of findings tables in systematic reviews are highly informative but require epidemiological training to be interpreted correctly. The usage of fishbone diagrams as graphical displays could offer researchers an effective approach to simplify content for readers with limited epidemiological training. In this paper we demonstrate how fishbone diagrams can be applied to systematic reviews and present the results of an initial user testing.

**Methods:**

Findings from two systematic reviews were graphically depicted in the form of the fishbone diagram. To test the utility of fishbone diagrams compared with summary of findings tables, we developed and pilot-tested an online survey using Qualtrics. Respondents were randomized to the fishbone diagram or a summary of findings table presenting the same body of evidence. They answered questions in both open-ended and closed-answer formats; all responses were anonymous. Measures of interest focused on first and second impressions, the ability to find and interpret critical information, as well as user experience with both displays. We asked respondents about the perceived utility of fishbone diagrams compared to summary of findings tables. We analyzed quantitative data by conducting t-tests and comparing descriptive statistics.

**Results:**

Based on real world systematic reviews, we provide two different fishbone diagrams to show how they might be used to display complex information in a clear and succinct manner. User testing on 77 students with basic epidemiological training revealed that participants preferred summary of findings tables over fishbone diagrams. Significantly more participants liked the summary of findings table than the fishbone diagram (71.8% vs. 44.8%; *p* < .01); significantly more participants found the fishbone diagram confusing (63.2% vs. 35.9%, *p* < .05) or indicated that it was difficult to find information (65.8% vs. 45%; p < .01). However, more than half of the participants in both groups were unable to find critical information and answer three respective questions correctly (52.6% in the fishbone group; 51.3% in the summary of findings group).

**Conclusions:**

Fishbone diagrams are compact visualizations that, theoretically, may prove useful for summarizing the findings of systematic reviews. Initial user testing, however, did not support the utility of such graphical displays.

**Electronic supplementary material:**

The online version of this article (10.1186/s12874-017-0452-z) contains supplementary material, which is available to authorized users.

## Background

Systematic reviews gather, describe, synthesize, and evaluate evidence on a wide range of topics in health care, many of which are complex and multi-faceted. The traditional approach to presenting such complexity is to develop multiple summary tables that describe the design of the studies, present results, and assess the quality of evidence. Such tables are often dense and do not allow readers to grasp the findings “at a glance” but instead require review of numerous pages of summary tables and large parts of the full evidence report [1].

Summary of findings tables such as those proposed by the Grading of Recommendations Assessment, Development and Evaluation (GRADE) Working Group [2], have become important tools to summarize results of systematic reviews and present the certainty of findings. They significantly improve readers’ overall understanding and their ability to find critical information compared with having data only in text [3].

Nevertheless, to be interpreted correctly, summary of findings tables require familiarity with some concepts of clinical epidemiology and with grading the certainty of the evidence. To address such limitations, a simplification of the content using graphical displays could provide an excellent approach for enhancing presentation to readers with limited epidemiological training [4].

**Fig. 1:**
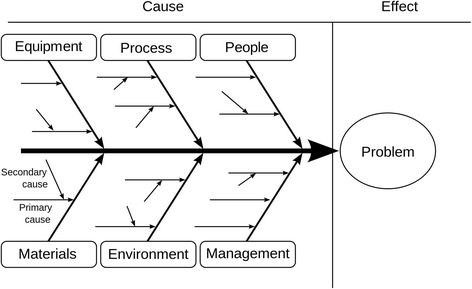
Example of a generic Ishikawa fishbone diagram https://commons.wikimedia.org/w/index.php?curid=6444290 (by Fabian Lange)

Fishbone diagrams were first proposed by Kaoro Ishikawa [5] in the 1960s to display cause and effect in the context of continuous improvement of industrial processes [6]. The diagram was first termed an “Ishikawa diagram” but was later dubbed “fishbone diagram” because of its resemblance to the skeleton of a fish—a horizontal spine with the “head” representing a problem or effect with the “bones” emanating at acute angles representing causes. Software for constructing fishbone diagrams is available within general graphics packages such as Microsoft Visio (Microsoft Corporation, Redmond, WA, USA) or in specific packages, such as SmartDraw© (https://www.smartdraw.com), which provides more flexibility for drawing, modifying, and annotating fishbone diagrams. Figure 1 presents a generic fishbone diagram.

Fishbone diagrams also have been widely used in health care for cause and effect analysis related to patient safety [7], and for evaluating quality management processes [8, 9].

To our knowledge, fishbone diagrams have not been used to summarize the findings of health care research. Currently no presentation tool exists that offers a less complex option than summary of findings tables yet more in-depth methodological information than fact boxes [10] that summarize the best available evidence on the benefits and harms of treatments but are targeted towards patients. Fishbone diagrams could possibly fulfill this need.

In this manuscript, we explore the utility of fishbone diagrams to display the totality of a body of evidence with multiple outcomes. We envision that fishbone diagrams could be effective tools for improving informed decision making among readers of systematic reviews with little epidemiological training. Theoretically, such a graphical technique could help readers to comprehend the conceptualization of the review and complex findings through a simple, succinct, and understandable format. Determining the best format of communicating risks and benefits of healthcare interventions is important because the correct interpretation of beneficial and harmful treatment effects is a prerequisite for informed decision making.

In the following sections, we first discuss the adaptation of the fishbone diagram using two real-world examples to show how systematic reviewers and others can use this graphical technique. Second, we summarize the results of a user testing exercise comparing a fishbone diagram with a GRADE summary of findings table.

## Methods

### Concept of the fishbone diagram to summarize evidence of systematic reviews

In our adaptation of the fishbone graphic, the “head” represents the overall balance between benefits and harms of an intervention or of competing interventions. The bones of the fish represent individual outcomes that are critical or important to balance the overall benefits and harms of the interventions. The proximity of the bones to the head reflects the importance of outcomes for decision making. GRADE, for example, recommends ranking the relative importance of outcomes when developing guidelines. Each bone (representing an outcome) can include additional explanatory factors such as a plain language summary of the effect, the number of studies, the magnitude of treatment effects, or other factors that influence the certainty (strength) of a body of evidence.

**Fig. 2:**
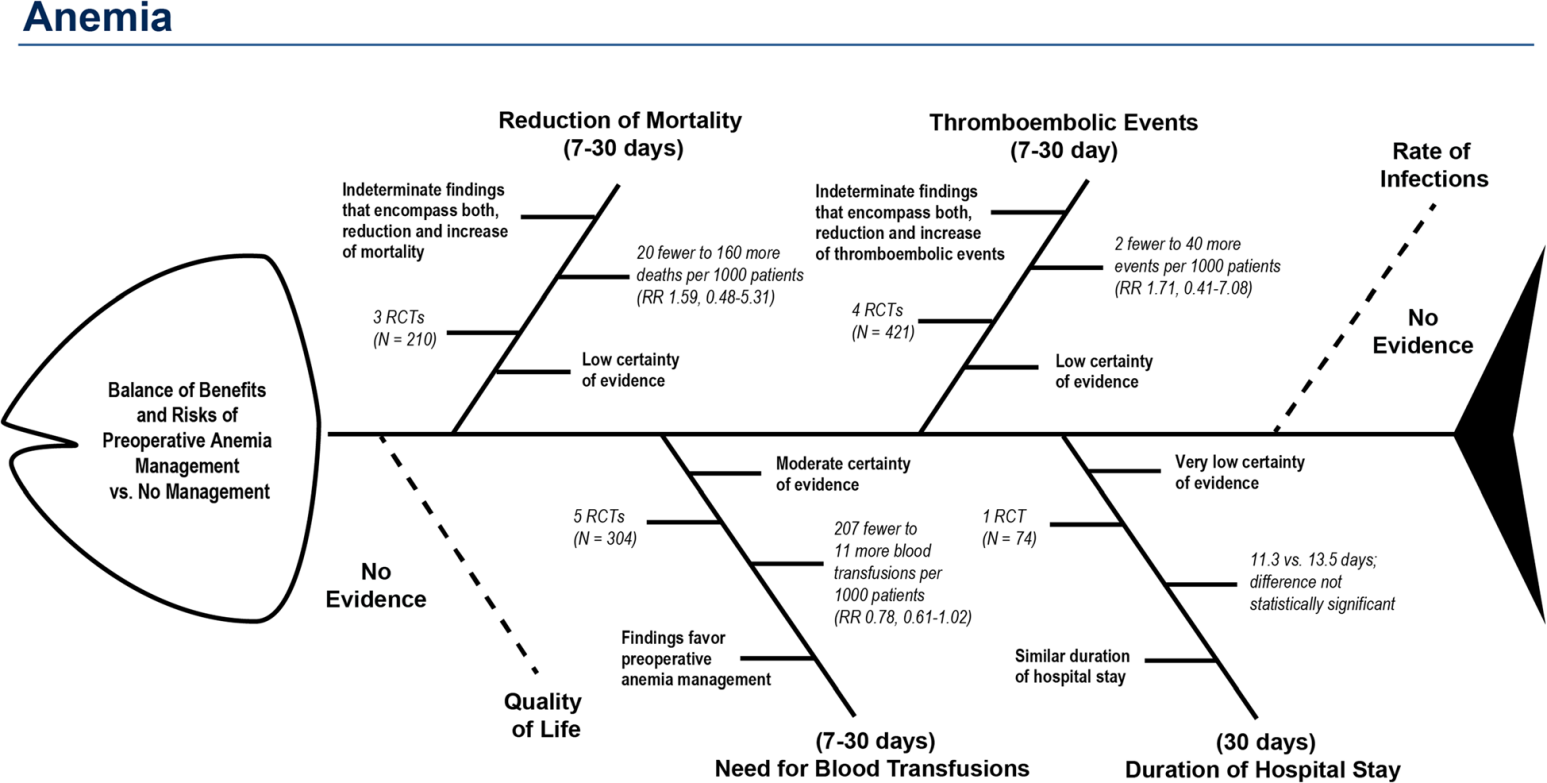
Fishbone diagram of benefits and risks of preoperative anemia management

**Fig. 3:**
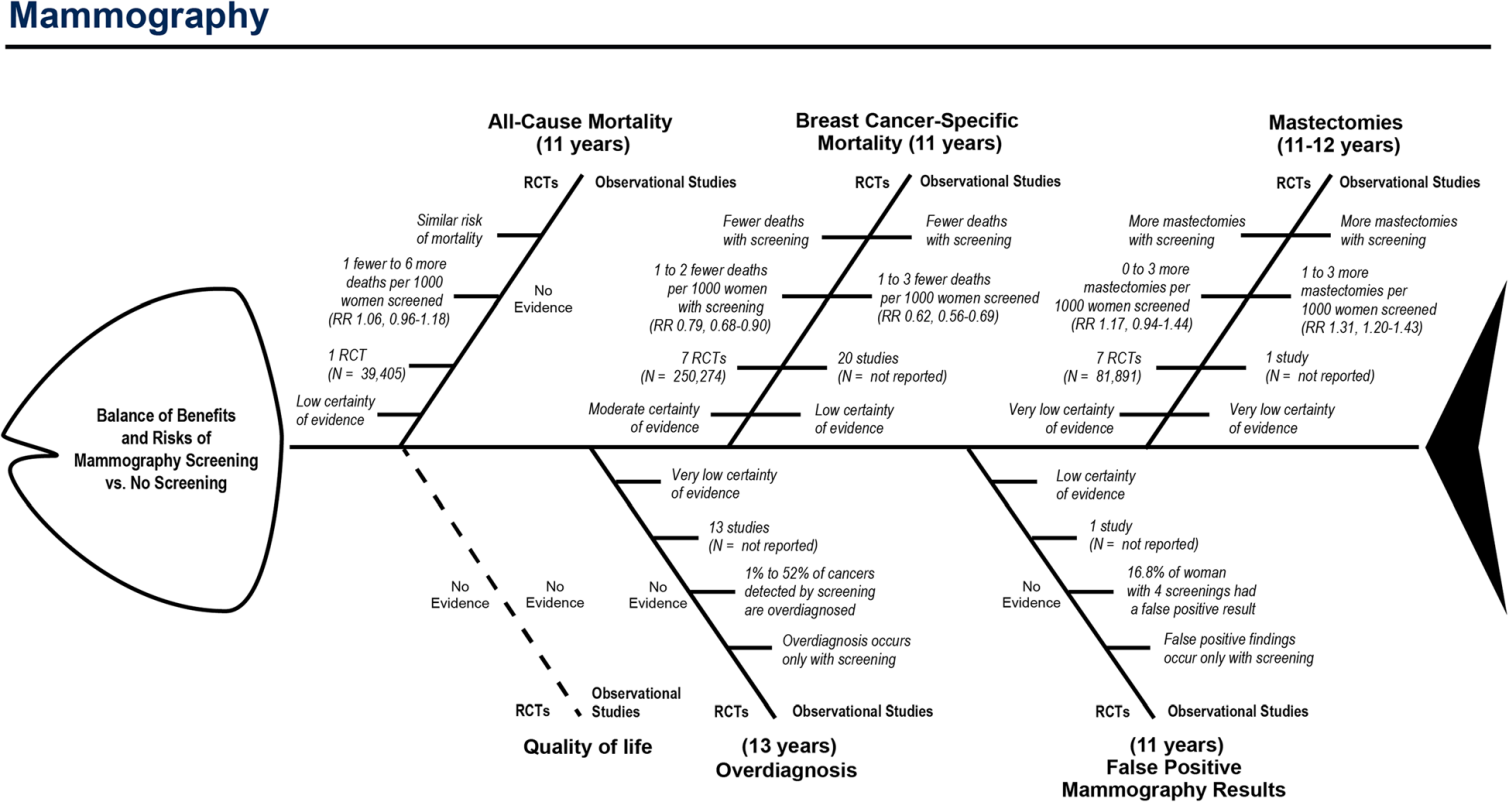
Fishbone diagram of benefits and risks of mammography screening vs. no screening

We applied the basic fishbone schematic to two systematic reviews [11, 12] that differed in key ways, so that we could illustrate the applications for various kinds of topics, patient populations, interventions, and outcomes. To understand whether the fishbone could usefully summarize comparative treatments that might form the basis of a clinical practice guideline, we took the findings from a systematic review of randomized controlled trials (RCTs) of preoperative anemia management versus usual care (“anemia fishbone”, Fig. 2) [11]. To evaluate the applicability and utility of fishbone graphics for preventive services, we selected an umbrella systematic review on the benefits and risks of mammography screening (“screening fishbone”, Fig. 3) [12].

For each fishbone diagram, we used and simplified information from the summary of findings table in each systematic review to populate the bones of the fish. One researcher populated each fishbone diagram initially; a second researcher evaluated the accuracy of the summary data. If discrepancies arose, the investigators arrived at a final version through consensus discussion or adjudication by a third researcher.

### Target audience

We envision as the target audience for fishbone diagrams, health professionals with some epidemiological training but who are not facile with comprehension of complex summary of findings tables.

### User testing

The goal of user testing was to compare the utility of a fishbone diagram to convey results of a systematic review with that of a GRADE summary of findings table. We conducted the user testing in English in January 2017 using Qualtrics, an electronic web-based survey tool. All answers were anonymous. Additional File 1 presents the structure of the survey as a flow diagram.

We pilot-tested the survey on health services researchers and revised the final version accordingly; the pilot data were not included in the final analyses. Except for initial impressions (see below) and demographic data, all items in the survey were in a forced-choice format. To allow for a mixed-methods approach in data analysis, we used both open-ended and closed answering formats. The Danube University Institutional Review Board determined that ethics approval was not required for anonymous user testing.

We used the “anemia fishbone” described above and presented in Fig. 2 as the example in the survey. The GRADE summary of findings table (Table 1) presented more detailed data than the fishbone diagram. Additional File 2 presents the final version of the survey.

**Table 1:** Summary of findings table of benefits and risks of preoperative anemia management

Outcomes	№ of participants (studies) Follow-up	Certainty of the evidence (GRADE)	Relative effect (95% CI)	Anticipated absolute effects	
				Risk with no intervention	Risk difference with preoperative treatment for anemia
Reduction of Mortality follow up: range 7 days to 30 days	210 (3 RCTs)	⨁⨁◯◯ LOW ^1^	RR 1.59 (0.48 to 5.31)	38 per 1000	23 more per 1000 (20 fewer to 160 more)
Quality of Life	(0 studies)	–	not estimable	0 per 1000	0 fewer per 1000 (0 fewer to 0 fewer)
Need for Blood Transfusions follow up: range 7 days to 30 days	304 (5 RCTs)	⨁⨁⨁◯ MODERATE ^2^	RR 0.78 (0.61 to 1.02)	532 per 1000	117 fewer per 1000(207 fewer to 11 more)
Duration of Hospital Stay follow up: mean 30 days	74 (1 RCT)	⨁◯◯◯ VERY LOW ^1,3^	not estimable	The mean duration of hospital stay was	11.3 vs. 13.5 days (difference not statistically significant)
Thromboembolic Events follow up: range 7 days to 30 days	421 (4 RCTs)	⨁⨁◯◯ LOW ^1^	RR 1.71 (0.41 to 7.08)	0 per 1000	0 fewer per 1000(2 fewer to 40 more)
Rate of Infections	(0 studies)	–	not estimable	0 per 1000	0 fewer per 1000 (0 fewer to 0 fewer)

*The risk in the intervention group (and its 95% confidence interval) is based on the assumed risk in the comparison group and the relative effect of the intervention (and its 95% CI)

CI: Confidence interval; RR: Risk ratio

GRADE Working Group grades of evidence

High certainty: We are very confident that the true effect lies close to that of the estimate of the effect

Moderate certainty: We are moderately confident in the effect estimate: The true effect is likely to be close to the estimate of the effect, but there is a possibility that it is substantially different

Low certainty: Our confidence in the effect estimate is limited: The true effect may be substantially different from the estimate of the effect

Very low certainty: We have very little confidence in the effect estimate: The true effect is likely to be substantially different from the estimate of effect

^1^ Few events, studies do not meet optimal information size, confidence intervals encompass clinically important differences

^2^ Studies do not meet optimal information size

^3^ High risk of bias of included trial

**Fig. 4:**
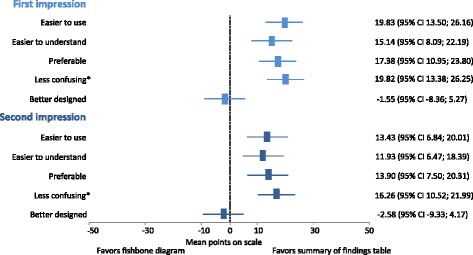
Direct comparison of fishbone diagrams with summary of findings tables before and after working with one of them

#### Sample

We used a nonrandom, purposive sample of students who were enrolled in a health sciences bachelor program or in a health management master’s program. All students had a basic understanding of clinical epidemiology. Because they had not been previously introduced to fishbone diagrams or summary of findings tables, we provided a brief introduction to the main concepts at the beginning of user testing. Respondents completed user testing via an online survey during regular class hours; however, participation was voluntary.

#### Measures

We were interested in four distinct measures for comparing the fishbone and the summary of findings table (Table 2): 1) Initial impressions; 2) Ability to find and interpret critical information; 3) Perceived utility; and 4) Second impression after using either the fishbone diagram or the summary of findings table.

**Table 2:** The ten most frequently named associations at first sight of the fishbone diagram and the summary of findings table

Fishbone Diagram		Summary of Findings Table	
Comment	Relative Frequency	Comment	Relative Frequency
Unclear	11.52%	structured	18.4%
Confusing	8.76%	clear	16.04%
Clear	5.99%	lots of information	10.38%
complicated	5.53%	common	6.13%
Chaotic	5.07%	complicated	6.13%
understandable	5.07%	boring	4.25%
Structured	4.61%	professional	3.77%
Creative	4.15%	unclear	2.83%
unstructured	4.15%	understandable	2.83%

To gather participants’ first impressions, we asked them to comment on their initial reactions when first viewing the two displays by writing down three spontaneous thoughts for the fishbone diagram and the summary of findings table. In addition, we had participants use a visual analog scale with a slider bar to compare the two displays based on five attributes *(‘easier to use’, ‘easier to understand’, ‘better designed’, ‘preferable’, ‘more confusing’*). The middle position indicated a neutral attitude. To continue with the questionnaire, the slider bars had to be clicked on by the participants and either moved or left in the neutral position.

To assess the ability to find and interpret critical information, we randomized participants to work either with the fishbone diagram or the summary of findings table. The participants were given a brief summary text that introduced the topic (preoperative anemia management) represented in the two displays. In a first step, participants had to choose the correct conclusion that could be drawn from the display (see Additional File 2 for details). In a second step, they had to answer three multiple-choice questions that required finding and interpreting data presented in the displays; only one answer per item was correct. Participants could also choose an “*I don’t know*” option. Because we were also interested in the participants’ speed of navigating the displays, we tracked the time from loading the page to providing the final answer for this section.

After having worked with either the fishbone diagram or the summary of findings table, participants were asked to evaluate the perceived utility of the respective display on several dimensions (e.g. ‘*By using the diagram, I can easily describe the risks and benefits of an intervention.*’) employing a six-point Likert scale from ‘*strongly disagree’ to ‘strongly agree*’.

To assess their “second impression” after working with one of the displays, participants again used a slider bar to compare the two displays based on the same five attributes as in the beginning of the survey (‘*easier to use’, ‘easier to understand’, ‘better designed’, ‘preferable’, ‘more confusing’*). We also asked participants which of the two displays they would recommend to a colleague, show to a patient, or suggest to researchers to summarize the results of systematic reviews.

#### Data analysis

Data were stored securely and were protected from unauthorized access. We analyzed quantitative data by conducting t-tests and comparing descriptive statistics in IBM SPSS Statistics Insert for Windows version 24 (IBM Corp. Armonk, New York, USA). To analyze qualitative data (participants’ first impressions), we first translated comments expressed in German into English and listed all data in Microsoft Excel, version 15.32 (Microsoft Corporation, Redmond Washington, USA). Then, we standardized synonyms and similar associations (e.g. ‘easy understanding’ and ‘easy to understand’ to ‘understandable’). Two raters carried out the translation and standardization of data consecutively and controlled each other’s work. Finally, we calculated the relative frequencies of associations.

## Results

We first illustrate the use of fishbone diagrams through the two topics described above. We then present results of the user testing based on the anemia fishbone.

### Example 1: Systematic review on preoperative anemia management

A professional society commissioned this systematic review as background for a panel developing clinical practice guidelines [11]. The anemia fishbone (Fig. 2) depicts the entire body of evidence from this review. The head of the fish represents the comparison of interest, namely the balance of benefits and risks of preoperative anemia management versus no management. Following the GRADE approach, the guideline panel selected six outcomes that they deemed *critical* or *important* for clinical decisionmaking; each bone represents an outcome. Critical outcomes are closer to the head than less important outcomes. A solid line between the fish spine and the outcome indicates that the review identified eligible evidence; dotted lines symbolize the lack of evidence (i.e., the review detected no eligible studies).

Each bone representing an outcome also provides a brief plain-language summary of the results and presents key characteristics of the evidence for that outcome: the number of trials, the total number of participants in these trials, the magnitude of effect in absolute and relative risks, and the certainty of evidence according to GRADE methods. For example, the panel ranked reduction of mortality and quality of life as critical outcomes, so both are close to the head. The evidence for reduced mortality consisted of three RCTs with a total of 210 participants. The pooled result of the three trials yielded an indeterminate effect estimate with wide confidence intervals including both benefits and harms (relative risk [RR] 1.59; 95% confidence interval [CI] 0.48, 5.31). The team that conducted the review graded the certainty of evidence as low. Because no evidence was available for quality of life, a dotted line is used. By contrast, the guideline panel rated the rate of infections and duration of hospital stay as important (but not critical) outcomes. Consequently, they are located close to the tail.

### Example 2: Umbrella systematic review on mammography screening

This example attempts to approximate the process of an international organization developing recommendations for mammography screening. Because of applicability concerns when relying on results only from RCTs, the guideline panel had commissioned a systematic review of systematic reviews (sometimes called an *umbrella review*) of both RCTs and observational studies [13].

The guideline panel had selected eight outcomes they deemed critical or important for decisionmaking. In our fishbone diagram, we depict the evidence for high-resource settings from both RCTs and observational studies in a parallel manner. By drawing the diagram this way, we can help users not only view important characteristics of each outcome but also compare findings on the same outcomes based on each body of evidence—RCTs and observational studies. Additional File 3 presents the corresponding summary of findings table.

### Results of user testing

The sample for user testing comprised 77 students. Most (75%) of the participants were female and were aged between 18 and 40 years (median = 22 years). All participants completed the survey. Additional File 4 presents supplementary information on user testing.

#### First impression

Participants’ first impressions of the displays favored summary of findings tables over fishbone diagrams. Out of 217 comments initially noted by participants, the most frequent associated with the fishbone diagram were *‘unclear’* (11.52%), *‘confusing’* (8.78%), and *‘clear’* (5.99%). From the 212 initial reactions for the summary of findings table, the most frequent ones were *‘structured’* (18.4%), *‘clear’* (16.04%), and ‘*lots of information’* (10.38%). Table 2 summarizes the ten most frequent comments for each display.

Figure 4 presents ratings of pre-determined attributes directly comparing the two displays after getting a first impression (light blue) and after having worked with either of the two displays (dark blue). Rectangles represent mean ratings. The middle position (0) indicates a neutral attitude. Overall, results favor the summary of findings table over the fishbone diagram and did not change substantially after having worked with either display. Additional File 4 presents box plots depicting the distribution of answers for each attribute.

#### Ability to find critical information

After participants had been randomized to either the fishbone diagram or the summary of findings table, they were asked to pick an overall conclusion and to answer three questions that required them to find and interpret facts from the displays (e.g., *Preoperative anemia management is favored the most by outcomes concerning: a) reduction of mortality, b) quality of life, c) need for blood transfusions, d) duration of hospital stay*). When asked to pick a correct overall conclusion, 68.4% of participants from the fishbone group and 71.8% from the summary of findings group provided the correct answer. Of those who picked the correct conclusion, participants in the fishbone group were on average 26 s faster in determining the correct answer (154.5 s vs. 180.8 s; difference not statistically significant). However, more than half of the participants in each group were unable to answer the three questions correctly that required finding and interpreting facts (52.6% in the fishbone group; 51.3% in the summary of findings group). Of those who picked the correct options, participants in the fishbone group were on average 26 s faster in deciding on the answers (123.2 s vs. 149.3 s) than those using the summary of findings table. The difference, however, was not statistically significant. More detailed results on the correct choice of options in each group can be found in Additional File 4.

#### Perceived utility

After working with the fishbone diagram or the summary of findings table, participants’ assessments of the perceived utility differed significantly between the two displays for some of the items. More participants using the summary of findings tables than the fishbone diagrams agreed with the statement ‘*Overall, I liked the diagram/table*’ (*p* < .01; combining *strongly agree/agree/slightly agree* options: 71.8% vs. 44.8%). By comparison, significantly more participants using the fishbone diagrams agreed with the statements ‘*It was hard to find the information I was interested in*’ (p < .01; combining *strongly agree/agree/slightly agree* options: 65.8% vs. 45%;) and ‘*The information in the diagram/table was confusing’* (*p* < .05; combining *strongly agree/agree/slightly agree* options: 63.2% vs. 35.9%).

Additional File 4 summarizes the perceived utility comparing fishbone diagrams with summary of findings tables. Overall, the perceived utility was better for summary of findings tables. Consequently, more participants would recommend summary of findings tables to a colleague (53.2% vs. 11.7%) or to a systematic reviewer (74.0% vs. 7.8%). Fewer participants, however, would recommend summary of findings tables than fishbone diagrams to patients (23.4% vs. 31.2%).

## Discussion

The concept of fishbone diagrams has been used in health care for decades for cause and effect analysis related to patient safety and for evaluating quality management processes. We presented two adaptations of a fishbone diagram to display large volumes of evidence in systematic reviews. The diagrams provide information-rich graphics that can supplement the complex and detailed tables that usually summarize the findings of systematic reviews. We envisioned fishbone diagrams as a useful tool for people with some epidemiological training but not enough familiarity with complex summary of findings tables.

User testing, however, revealed that students with basic training in clinical epidemiology found fishbone diagrams complicated and difficult to understand. Participants rated the perceived utility of summary of findings tables as better than that of fishbone diagrams. A somewhat surprising result, however, was that more than half of the participants in each group were unable to answer all three factual questions correctly. This finding confirms that summary of findings tables, despite their widespread use, are not an ideal way to convey key points about evidence to readers of systematic reviews with little epidemiological training.

An important reason for the unfavorable results concerning fishbone diagrams is probably that most participants were confronted with this concept for the first time. We briefly explained the basic idea of fishbone diagrams before user testing but this could probably not overcome the fact that participants were more familiar with tables. The unfavorable ratings, however, pertain to first impressions and the perceived utility only. When we tested how well participants can detect and interpret facts presented in fishbone diagrams or summary of findings tables, no substantial differences were identified.

Our user testing has several limitations. First, by surveying students unfamiliar with the concept of fishbone diagrams, we probably placed fishbone diagrams at a disadvantage compared with summary of findings tables. Had participants received some training in the usage of fishbone diagrams, the perceived utility might have been better. We did not provide training so as to emulate usual situations in which systematic reviews are used. The average reader who might confront a fishbone diagram in a systematic review would likely lack training in the diagram usage. Second, the participants of user testing comprised of students who had at least one course in clinical epidemiology and evidence-based medicine. Clinicians and decision-makers who read systematic reviews might not have this background knowledge. The applicability of our findings to other populations with different educational backgrounds, therefore, is unclear. Third, we conducted the pilot test in health services researchers, some of whom had many years of experience with conducting and interpreting evidence syntheses. The fact that the pilot population had more experience with evidence syntheses than the actual population in the user tests could have contributed to a potential lack of understanding of the purpose of the user test. We cannot rule out a certain extent of measurement error because participants may have misunderstood our questions.

Despite the unfavorable results of the user test, we still believe that fishbone diagrams, in general, have the potential to convey the facts of systematic reviews in a structured and efficient manner. The fishbone diagram is a flexible tool; users can adapt it in numerous ways based on the details of the systematic review or the needs of the users (e.g., guideline developers, clinicians). For example, by juxta-positioning results from RCTs and observational studies in our mammography fishbone diagram, we allow for a quick comparison of effects from RCTs and observational studies which can help readers gauge potential issues with applicability of results from RCTs. With its easily modifiable structure, the number of bones for any given systematic review topic can be increased or reduced depending on whether the focus is narrowed or expanded. Additionally, researchers can experiment with and test other graphical elements to characterize numerous pieces of information, such as the types of studies summarized, the quality of the evidence, or the magnitude of the overall effect. Such elements can include color, line width (varying thicknesses), and line type (e.g., solid or dotted). The time investment to create fishbone diagrams is comparable to the time to create a summary of findings table, particularly when an existing template can be used. Compared with summary of findings tables, however, the space to present results in fishbone diagrams is limited. Outcomes on benefits and harms, therefore, need to be limited to those that are most relevant to patients and most important for decisionmaking. The GRADE Working Group, for example, recommends limiting the number of outcomes to no more than seven [14], an amount that can be easily accommodated within a fishbone diagram.

Clearly, further work is needed to enhance the usefulness of fishbone diagrams for the field of evidence-based practice and systematic reviews. In much the same way that survey instruments or health communications techniques are tested through focus groups and cognitive interviewing, different kinds of fishbone diagrams may usefully be examined through these techniques. Finding the most comprehensible presentation formats for specific target groups is crucial because fully understanding the magnitude of benefits and risks is the prerequisite for any informed decision.

## Conclusions

Initial user testing of fishbone diagrams as compact visualizations for presenting the essence of a systematic review did not support the utility of such graphical displays. The low proportions of correct answers of users of fishbone diagrams and summary of findings tables, however, indicate a general problem with the effectiveness of conveying key points about evidence in systematic reviews.

## Additional files

Additional file 1: Flow chart. Flow Diagram of Survey Word File (figure) (DOCX 26 kb)

Additional file 2: Survey. Survey Word File (DOCX 29 kb)

Additional file 3: Mammography. Summary of Findings Table for Mammography Screening Word File (DOCX 51 kb)

Additional file 4: Supplementary Results. Supplementary Results of User Testing Word File (DOCX 107 kb)

## Abbreviations

CI: Confidence interval
RCT: Randomized controlled trial
RR: Relative risk
