# Supplementary material for: User testing of an adaptation of fishbone diagrams to depict results of systematic reviews
Source: BMC Med Res Methodol. 2017 Dec 12;17:169. doi: 10.1186/s12874-017-0452-z (PMC5727698; doi:10.1186/s12874-017-0452-z)
Supplement: Supplementary file 1 — Flow chart. Flow Diagram of Survey Word File (figure) (DOCX 26 kb) [file 12874_2017_452_MOESM1_ESM.docx]

**Additional File 1: Flow Diagram of Survey**

Data collection:

*Ability to find and interpret critical information*

*Perceived utility*

Data collection:

*Ability to find and interpret critical information*

*Perceived utility*

Data collection: *First impression*

## Third Part

## Second Part

## First Part

## Allocation

Allocated to **summary of findings table**

(n= 39)

Analysed (n= 77)
Excluded from analysis (n= 0)

Data collection: *Second impression*

Excluded (n= 0)

Allocated to **fishbone diagram** (n= 38)

Enrolled (n= 77)

Randomized (n= 77)
