# Supplementary material for: User testing of an adaptation of fishbone diagrams to depict results of systematic reviews
Source: BMC Med Res Methodol. 2017 Dec 12;17:169. doi: 10.1186/s12874-017-0452-z (PMC5727698; doi:10.1186/s12874-017-0452-z)
Supplement: Supplementary file 2 — Survey. Survey Word File (DOCX 29 kb) [file 12874_2017_452_MOESM2_ESM.docx]

**Additional File 2: Survey**

Fishbone Testing, Jan 2017

Q1.1 Welcome!  Thank you very much for participating in our survey. We are interested in your opinion about two different forms of illustrating results of systematic reviews. All answers are anonymous. We will analyze all data only in an aggregated manner. Cochrane Austria at the Danube University Krems is conducting this survey in cooperation with RTI International, North Carolina, USA. The survey takes approximately 20-25 minutes. Please continue to the next page and complete the exercises and related survey questions on each page. Please be aware that it is not possible to go back to a previous page once you have continued to the next page.

Q2.1 Below you can see two diagrams presenting the available evidence on benefits and harms of pre-operative anemia management compared with usual care (i.e., no active anemia management). Patients with anemia who undergo surgery have a higher risk for intra-operative and post-operative complications. Pre-operative anemia management describes diagnostic and therapeutic procedures in advance of a scheduled surgery to detect and treat anemia before patients undergo surgery.Both diagrams summarize the same facts. One is a Fishbone Diagram; the other is a GRADE (Grading of Recommendations Assessment, Development and Evaluation) Summary of Findings Table. Review each diagram by clicking on them individually to view the larger version. The images will be opened in a separate tab. Then, below, tell us in your own words what your first impressions of each diagram were.

Q2.2     Click on each image to enlarge

Q2.3 Please name up to three attributes that come to mind when looking at the diagrams:  Fishbone Diagram

1 (1)

2 (2)

3 (3)

Q2.4 Summary of Findings Table

1 (1)

2 (2)

3 (3)

Q2.5 Timing

First Click (1)

Last Click (2)

Page Submit (3)

Click Count (4)

Q2.6     Click on each image to enlarge

Q2.7 Please assign the following attributes to either the Fishbone Diagram or the Summary of Findings Table. The more applicable you find the attribute to the Fishbone Diagram, the further you move the slider bar to the left side. The more applicable you find the attribute to the Summary of Findings Table, the further you move it to the right side. Which one of these ways of presenting information is…

______ Easier to use (1)

______ Easier to understand (2)

______ Better designed (3)

______ Preferable (4)

______ More confusing (5)

Q2.8 Timing

First Click (1)

Last Click (2)

Page Submit (3)

Click Count (4)

Q3.1 You were randomly assigned to work with the Fishbone Diagram. Let us explain the structure of the diagram: The question of interest is stated in the head. Each main bone represents an outcome of interest, such as reduction of mortality or quality of life. The proximity of the bone to the head indicates the importance of the outcome for decision making — the nearer to the head, the greater the importance. For each outcome of interest, the side bones that come off the main bone summarize the evidence, the certainty of these findings, the number of studies and participants, and the effect estimates in absolute and relative measures.   Click on image to enlarge

Q3.2 Which of the following sets of conclusions can you draw from the diagram?

- According to the diagram, preoperative anemia management reduces the need for blood transfusions, does not shorten the duration of hospital stays significantly and no evidence is available about the impact on quality of life. (1)
- According to the diagram, following preoperative anemia management, more deaths occur because of thromboembolic events, there is a lower rate of infections and a longer duration of hospital stays. (2)
- According to the diagram, preoperative anemia management causes a higher need for blood transfusions, an increase in mortality and an increase in thromboembolic events. (3)
- I don’t know. (4)

Q3.3 Timing

First Click (1)

Last Click (2)

Page Submit (3)

Click Count (4)

Q3.4 Click on image to enlarge

Q3.5 Based on information in the diagram, which facts are correct?

Q3.6 Conclusions on the need for blood transfusions following preoperative anemia management are based on studies with a total of

- 304 patients (1)
- 207 patients (2)
- 1000 patients (3)
- 5 patients (4)
- I don’t know (5)

Q3.7 Preoperative anemia management is only favored by findings concerning

- mortality (1)
- quality of life (2)
- need for blood transfusions (3)
- duration of hospital stay (4)
- I don’t know (5)

Q3.8 For mortality, the relative risk (RR) of pre-operative anemia management vs. no management is

- RR 1.71 (1)
- RR 0.78 (2)
- RR 1.59 (3)
- RR 11.3 (4)
- I don't know (5)

Q3.9 Timing

First Click (1)

Last Click (2)

Page Submit (3)

Click Count (4)

Q3.10 Please indicate your personal opinion about the diagram.

Q3.11 Click on image to enlarge

Q3.12 For each of the statements below, please select one option indicating how strongly you agree or disagree.

|  | Strongly Disagree (1) | Disagree (2) | Slightly Disagree (3) | Slightly Agree (4) | Agree (5) | Strongly Agree (6) |
| --- | --- | --- | --- | --- | --- | --- |
| Overall, I liked the diagram. (1) |  |  |  |  |  |  |
| I liked the way the information was organized. (2) |  |  |  |  |  |  |
| It was hard to find the information I was interested in. (3) |  |  |  |  |  |  |
| The information in the diagram was confusing. (4) |  |  |  |  |  |  |
| By using the diagram, I can easily describe the risks and benefits of an intervention. (5) |  |  |  |  |  |  |

Q3.13 Please record any further comments you might have about the diagram:

Q3.14 Timing

First Click (1)

Last Click (2)

Page Submit (3)

Click Count (4)

Q4.1   You were randomly assigned to work with the Summary of Findings Table. Let us explain the structure of this table: The question of interest is stated at the top of the table Each row presents an outcome of interest, such as mortality or quality of life Outcomes appearing closer to the top of the table are more important for decision making than outcomes toward the bottom For each outcome, the rows present the number of studies and participants, the effect estimates in absolute and relative measures, and the certainty of the evidence​ Click on image to enlarge

Q4.2 Which of the following sets of conclusions can you draw from the table?

- According to the table, preoperative anemia management reduces the need for blood transfusions, does not shorten the duration of hospital stays significantly and no evidence is available about the impact on quality of life. (1)
- According to the table, following preoperative anemia management, more deaths occur because of thromboembolic events, there is a lower rate of infections and a longer duration of hospital stays. (2)
- According to the table, preoperative anemia management causes a higher need for blood transfusions, an increase in mortality and an increase in thromboembolic events. (3)
- I don’t know. (4)

Q4.3 Timing

First Click (1)

Last Click (2)

Page Submit (3)

Click Count (4)

Q4.4 Click on image to enlarge

Q4.5 Based on information in the table, which facts are correct?

Q4.6 Conclusions on the need for blood transfusions following preoperative anemia management are based on studies with a total of

- 304 patients (1)
- 207 patients (2)
- 1000 patients (3)
- 5 patients (4)
- I don’t know (5)

Q4.7 Preoperative anemia management is only favored by findings concerning

- mortality (1)
- quality of life (2)
- need for blood transfusions (3)
- duration of hospital stay (4)
- I don’t know (5)

Q4.8 For mortality, the relative risk (RR) of pre-operative anemia management vs. no management is

- RR 1.71 (1)
- RR 0.78 (2)
- RR 1.59 (3)
- RR 11.3 (4)
- I don't know (5)

Q4.9 Timing

First Click (1)

Last Click (2)

Page Submit (3)

Click Count (4)

Q4.10 Please indicate your personal opinion about the table.

Q4.11 Click on image to enlarge

Q4.12 For each of the statements below, please select one option indicating how strongly you agree or disagree.

|  | Strongly Disagree (1) | Disagree (2) | Slightly Disagree (3) | Slightly Agree (4) | Agree (5) | Strongly Agree (6) |
| --- | --- | --- | --- | --- | --- | --- |
| Overall, I liked the table. (1) |  |  |  |  |  |  |
| I liked the way the information was organized. (2) |  |  |  |  |  |  |
| It was hard to find the information I was interested in. (3) |  |  |  |  |  |  |
| The information in the table was confusing. (4) |  |  |  |  |  |  |
| By using the table, I can easily describe the risks and benefits of an intervention. (5) |  |  |  |  |  |  |

Q4.13 Please record any further comments you might have about the table:

Q4.14 Timing

First Click (1)

Last Click (2)

Page Submit (3)

Click Count (4)

Q5.1 Now that you have worked with either the Fishbone Diagram or the Summary of Findings Table, please review both of them again and provide your assessments about the attributes listed below.     Click on an image to enlarge

Q5.2 Which one of these ways of presenting information is …

______ Easier to use? (1)

______ Easier to understand? (2)

______ Better designed? (3)

______ Preferable? (4)

______ More confusing? (5)

Q5.3 Which one of these ways of presenting information would you recommend to a colleague?

- The Fishbone Diagram (1)
- The Summary of Findings Table (2)
- Both (3)
- Neither of them (4)

Q5.4 Which one of these ways of presenting information would you show to a patient?

- The Fishbone Diagram (1)
- The Summary of Findings Table (2)
- Both (3)
- Neither of them (4)

Q5.5 Which one of these ways of presenting information would you recommend to researchers to summarize the results of a systematic review

- The Fishbone Diagram (1)
- The Summary of Findings Table (2)
- Both (3)
- Neither of them (4)

Q5.6 Timing

First Click (1)

Last Click (2)

Page Submit (3)

Click Count (4)

Q6.1 What is your age? (e.g. "25")

Q6.2 You are:

- Female (1)
- Male (2)

Q6.3 How long have you been enrolled in your current study program?

- 1 semester (1)
- 2 semesters (2)
- 3 semesters (3)
- 4 semesters (4)
- 5 semesters (5)
- 6 semesters (6)
- 7 semesters (7)
- 8 semesters (8)
- 9 or more semesters (9)
