# Supplementary material for: User testing of an adaptation of fishbone diagrams to depict results of systematic reviews
Source: BMC Med Res Methodol. 2017 Dec 12;17:169. doi: 10.1186/s12874-017-0452-z (PMC5727698; doi:10.1186/s12874-017-0452-z)
Supplement: Supplementary file 3 — Mammography. Summary of Findings Table for Mammography Screening Word File (DOCX 51 kb) [file 12874_2017_452_MOESM3_ESM.docx]

**Additional File 3: Summary of Findings Table for Mammography Screening**

| **Outcomes** | **№ of participants (studies) Follow-up** | **Quality of the evidence (GRADE)** | **Relative effect (95% CI)** | **Anticipated absolute effects** | |
| --- | --- | --- | --- | --- | --- |
|  |  |  |  | **Risk with no Screening** | **Risk difference with**  **Screening** |
| Breast Cancer Specific Mortality-RCTs follow up: mean 11 years | 250,274 (7 RCTs) | ⨁⨁⨁◯ MODERATE^1^ | **RR 0.79** (0.68 to 0.90) | 6 per 1000 | **1 fewer per 1000** (1 fewer to 2 fewer) |
| Breast Cancer Specific Mortality-observational studies follow up: mean 11 years | not reported (20 observational studies) | ⨁⨁◯◯ LOW | **RR 0.62** (0.56 to 0.69) | 6 per 1000 | **2 fewer per 1000** (1 fewer to 3 fewer) |
| Mastectomies-RCTs follow up: mean 11 years | 81,891 (7 RCTs) | ⨁◯◯◯ VERY LOW ^1,2^ | **RR 1.17** (0.94 to 1.44) | 6 per 1000 | **1 more per 1000** (0 fewer to 3 more) |
| Mastectomies-observational studies follow up: mean 12 years | not reported (1 observational study) | ⨁◯◯◯ VERY LOW ^3^ | **RR 1.31** (1.20 to 1.43) | 6 per 1000 | **2 more per 1000** (1 more to 3 more) |
| Overdiagnosis-RCTs | (0 RCTs) | - | not estimable | not estimable | not estimable |
| Overdiagnosis-observational studies follow up: mean 13 years | not reported (13 observational studies) | ⨁◯◯◯ VERY LOW | not estimable | not estimable | **not estimable**; 1% to 52% of cancers detected by screening are estimated to be due to overdiagnosis |
| False Positive Mammography Results-RCTs | (0 RCTs) | - | not estimable | 0 per 1000 | **0 fewer per 1000** (0 fewer to 0 fewer) |
| False Positive Mammography Results-observational studies follow up: mean 11 years | not reported (1 observational study) | ⨁⨁◯◯ LOW | not estimable | not estimable | **not estimable;** 16.8% of women after 4 screening rounds had a false positive result |
| All cause mortality-RCTs  Follow up: mean 11 years | 39,405 (1 RCT) | ⨁⨁◯◯ LOW ^4^ | RR 1.06 (0.96 to 1.18) | 35 per 1000 | **2 more per 1000** (1 fewer to 6 more) |
| All-cause mortality -observational studies | (0 observational studies) | - | not estimable | 0 per 1000 | **0 fewer per 1000** (0 fewer to 0 fewer) |
| Quality of Life-RCTs | (0 RCTs) | - | not estimable | 0 per 1000 | **0 fewer per 1000** (0 fewer to 0 fewer) |
| Quality of Life-observational studies | (0 observational studies) | - | not estimable | 0 per 1000 | **0 fewer per 1000** (0 fewer to 0 fewer) |

| ***The risk in the intervention group** (and its 95% confidence interval) is based on the assumed risk in the comparison group and the **relative effect** of the intervention (and its 95% CI).  **CI:** Confidence interval; **RR:** Risk ratio |
| --- |
| **GRADE Working Group grades of evidence** **High quality:** We are very confident that the true effect lies close to that of the estimate of the effect **Moderate quality:** We are moderately confident in the effect estimate: The true effect is likely to be close to the estimate of the effect, but there is a possibility that it is substantially different **Low quality:** Our confidence in the effect estimate is limited: The true effect may be substantially different from the estimate of the effect **Very low quality:** We have very little confidence in the effect estimate: The true effect is likely to be substantially different from the estimate of effect |

Explanations

^1^. High risk of bias of several included trials

^2^. Confidence interval crosses threshold of appreciable harm; studies do not meet optimal informations size

^3^. Studies do not meet optimal information size

^4^. Confidence interval crosses threshold of appreciable harm
