# Supplementary material for: User testing of an adaptation of fishbone diagrams to depict results of systematic reviews
Source: BMC Med Res Methodol. 2017 Dec 12;17:169. doi: 10.1186/s12874-017-0452-z (PMC5727698; doi:10.1186/s12874-017-0452-z)
Supplement: Supplementary file 4 — Supplementary Results. Supplementary Results of User Testing Word File (DOCX 107 kb) [file 12874_2017_452_MOESM4_ESM.docx]

**Additional File 4: Supplementary Results of User Testing**

**4.1 First and second impressions**

Figure 1: Box plots representing the distribution of answers of participants regarding attributes comparing fishbone diagrams and summary of findings tables (depicted in Figure 4)

1. Please assign the following attributes to either the Fishbone Diagram or the Summary of Findings...-Easier to use


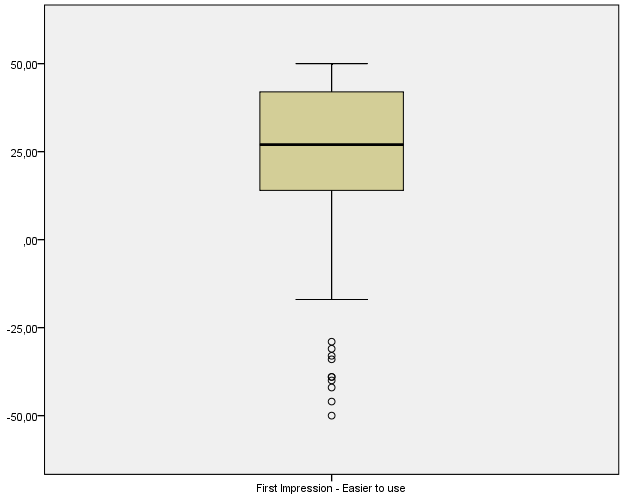


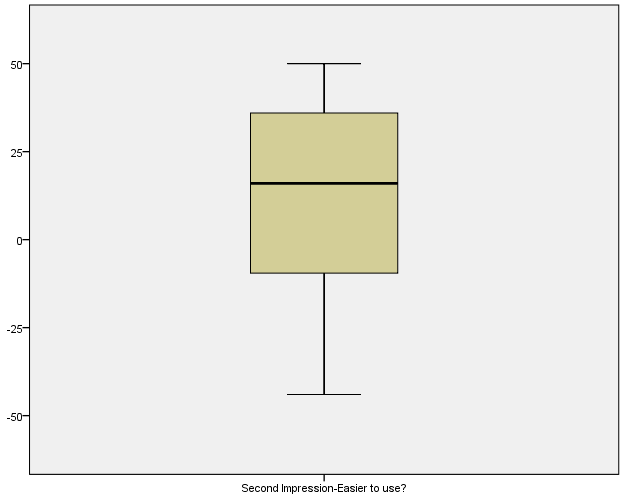


2. Please assign the following attributes to either the Fishbone Diagram or the Summary of Findings...-Easier to understand


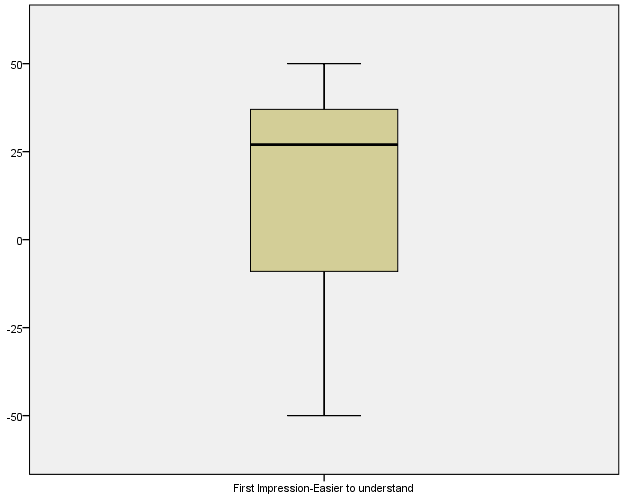


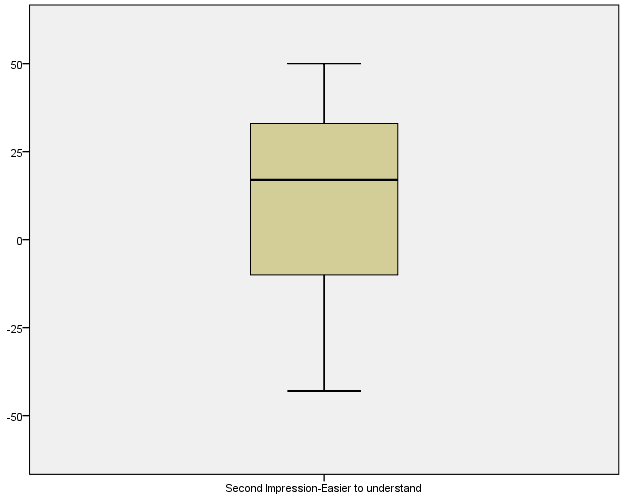


3. Please assign the following attributes to either the Fishbone Diagram or the Summary of Findings...-Preferable


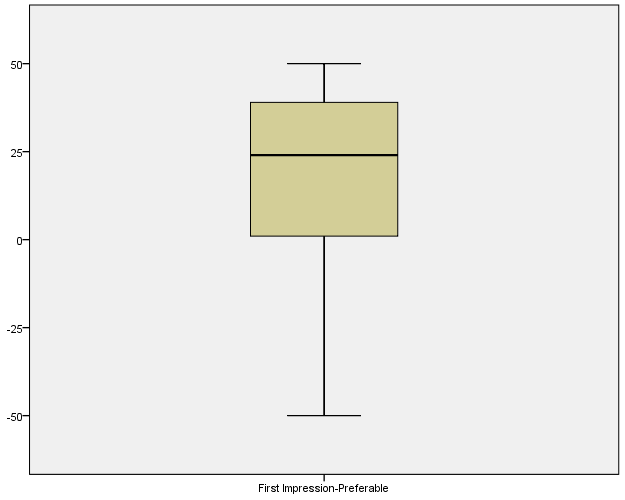

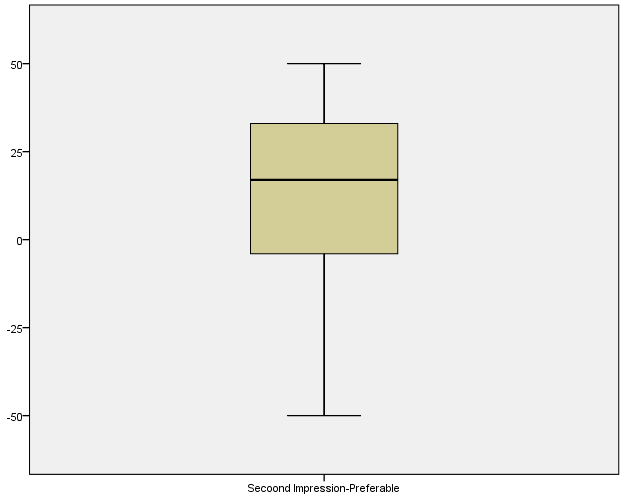


4. Please assign the following attributes to either the Fishbone Diagram or the Summary of Findings...-More confusing


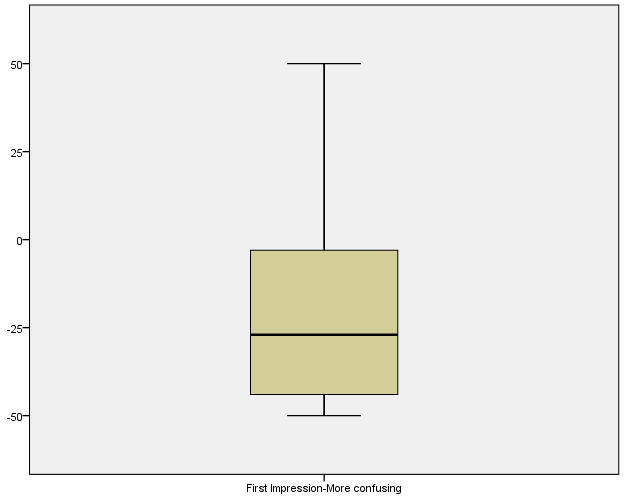

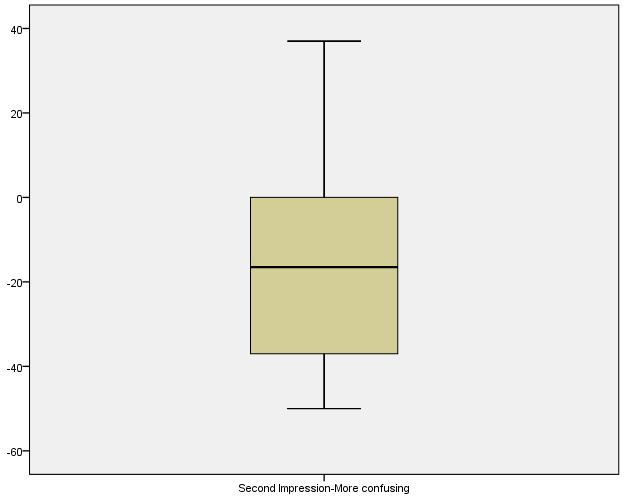


5. Please assign the following attributes to either the Fishbone Diagram or the Summary of Findings...-Better designed


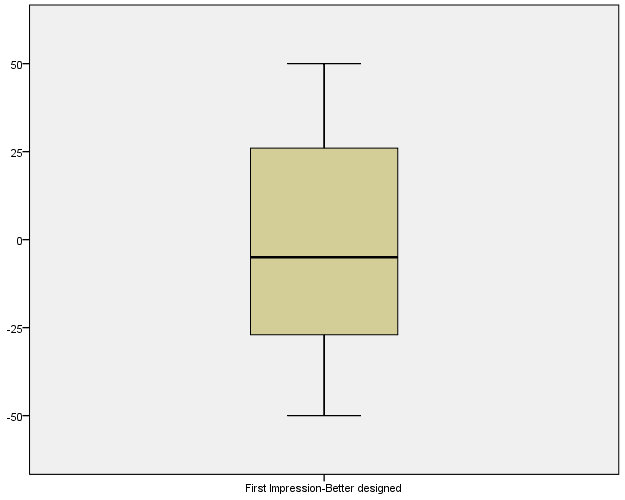

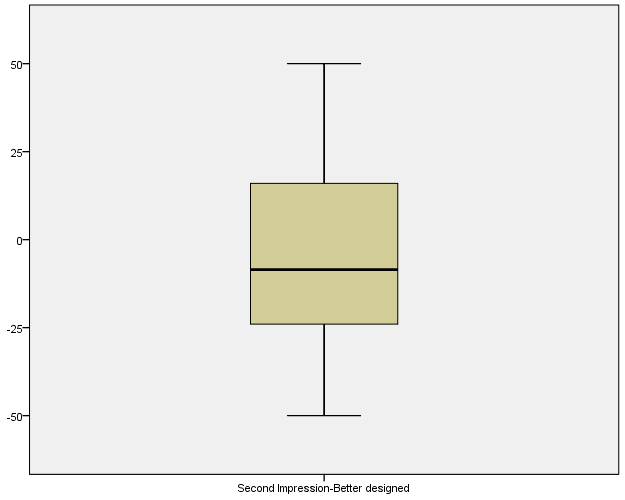


**4.2 Ability to find critical information**

Table 1**:** Percentage of participants selecting different overall conclusions based on either the fishbone diagram or the summary of findings table (the correct answer is bold).

| Set of general conclusions | Fishbone group (n=38) | Summary of findings table group (n=39) |
| --- | --- | --- |
| According to the diagram/table, preoperative anemia management reduces the need for blood transfusions, does not shorten the duration of hospital stays significantly and there is no evidence about the impact on quality of life. | **68.4%** | **71.8%** |
| According to the diagram/table, following preoperative anemia management there are more deaths due to thromboembolic events, a lower rate of infections and a longer duration of hospital stays. | 7.9% | 5.1% |
| According to the diagram/table, the risks of preoperative anemia management include a higher need for blood transfusions, an increase of mortality and thromboembolic events. | 10.5% | 10.3% |
| I don’t know. | 13.2% | 12.8% |

Table 2**:** Percentage of participants selecting specific facts based on either the fishbone diagram or the summary of findings table (correct answers are bold).

| Item | | Fishbone group (n=38) | Summary of findings table group (n=39) |
| --- | --- | --- | --- |
| Conclusions on the need for blood transfusions following preoperative anemia management are based on studies featuring | **304 patients** | **63.2%** | **79.5%** |
|  | 207 patients | 7.9% | 0% |
|  | 1000 patients | 23.7% | 15.4% |
|  | 5 patients | 0% | 0% |
|  | I don’t know | 5.3% | 5.1% |
| Preoperative anemia management is only favored by findings concerning | mortality | 10.5% | 5.1% |
|  | quality of life | 7.9% | 5.1% |
|  | **need for blood transfusions** | **73.7%** | **69.2%** |
|  | duration of hospital stay | 2.6% | 7.7% |
|  | I don’t know | 5.3% | 12.8% |
| For mortality, the relative risk (RR) of pre-operative anemia management vs. no management is | RR 1.71 | 0% | 0% |
|  | RR 0.78 | 7.9% | 0% |
|  | **RR 1.59** | **84.2%** | **92.3%** |
|  | RR 11.3 | 0% | 2.6% |
|  | I don’t know | 7.9% | 5.1% |

**4.3 Perceived utility**

Table 3: Perceived utility comparing fishbone diagrams with summary of findings tables.

| Item | | Fishbone group (n=38) | Summary of findings table group (n=39) |
| --- | --- | --- | --- |
| Overall, I liked the diagram/table. | strongly agree | 0% | 5.1% |
|  | agree | 31.6% | 46.2% |
|  | slightly agree | 13.2% | 20.5% |
|  | slightly disagree | 10.5% | 20.5% |
|  | disagree | 36.8% | 2.6% |
|  | strongly disagree | 7.9% | 5.1% |
| I liked the way the information was organized. | strongly agree | 10.5% | 7.7% |
|  | agree | 21.1% | 28.2% |
|  | slightly agree | 18.4% | 33.3% |
|  | slightly disagree | 15.8% | 23.1% |
|  | disagree | 28.9% | 7.7% |
|  | strongly disagree | 5.3% | 0% |
| It was hard to find the information I was interested in. | strongly agree | 7.9% | 2.6% |
|  | agree | 39.5% | 12.8% |
|  | slightly agree | 18.4% | 25.6% |
|  | slightly disagree | 10.5% | 17.9% |
|  | disagree | 23.7% | 30.8% |
|  | strongly disagree | 0% | 10.3% |
| The information in the diagram/table was confusing. | strongly agree | 2.6% | 7.7% |
|  | agree | 47.4% | 5.1% |
|  | slightly agree | 13.2% | 23.1% |
|  | slightly disagree | 7.9% | 15.4% |
|  | disagree | 23.7% | 38.5% |
|  | strongly disagree | 5.3% | 10.3% |
| By using the diagram/table, I can easily describe the risks and benefits of an intervention. | strongly agree | 0% | 2.6% |
|  | agree | 28.9% | 20.5% |
|  | slightly agree | 13.2% | 38.5% |
|  | slightly disagree | 15.8% | 12.8% |
|  | disagree | 26.3% | 20.5% |
|  | strongly disagree | 15.8% | 5.1% |
